# Interaction between host genes and *Mycobacterium tuberculosis* lineage can affect tuberculosis severity: Evidence for coevolution?

**DOI:** 10.1371/journal.pgen.1008728

**Published:** 2020-04-30

**Authors:** Michael L. McHenry, Jacquelaine Bartlett, Robert P. Igo, Eddie M. Wampande, Penelope Benchek, Harriet Mayanja-Kizza, Kyle Fluegge, Noemi B. Hall, Sebastien Gagneux, Sarah A. Tishkoff, Christian Wejse, Giorgio Sirugo, W. Henry Boom, Moses Joloba, Scott M. Williams, Catherine M. Stein

**Affiliations:** 1 Department of Population and Quantitative Health Sciences, Case Western Reserve University School of Medicine, Cleveland, Ohio, United States of America; 2 Department of Medical Microbiology, College of Health Sciences, Makerere University, Kampala, Uganda; 3 Department of Medicine and Mulago Hospital, School of Medicine, Makerere University, Kampala, Uganda; 4 Swiss Tropical and Public Health Institute, Basel, Switzerland; 5 University of Basel, Basel, Switzerland; 6 Departments of Genetics and Biology, University of Pennsylvania, Philadelphia, Pennsylvania, United States of America; 7 Department of Infectious Diseases and Center for Global Health, Aarhus University, Aarhus, Denmark; 8 Bandim Health Project, INDEPTH Network, Bissau, Guinea Bissau; 9 Department of Systems Pharmacology and Translational Therapeutics, University of Pennsylvania, Philadelphia, Pennsylvania, Unites States of America; 10 Tuberculosis Research Unit, Case Western Reserve University School of Medicine, Cleveland, Ohio, United States of America; 11 Department of Genetics and Genome Sciences, Case Western Reserve University School of Medicine, Cleveland, Ohio, United States of America; McGill University, CANADA

## Abstract

Genetic studies of both the human host and *Mycobacterium tuberculosis* (MTB) demonstrate independent association with tuberculosis (TB) risk. However, neither explains a large portion of disease risk or severity. Based on studies in other infectious diseases and animal models of TB, we hypothesized that the genomes of the two interact to modulate risk of developing active TB or increasing the severity of disease, when present. We examined this hypothesis in our TB household contact study in Kampala, Uganda, in which there were 3 MTB lineages of which L4-Ugandan (L4.6) is the most recent. TB severity, measured using the Bandim TBscore, was modeled as a function of host SNP genotype, MTB lineage, and their interaction, within two independent cohorts of TB cases, N = 113 and 121. No association was found between lineage and severity, but association between multiple polymorphisms in IL12B and TBscore was replicated in two independent cohorts (most significant rs3212227, combined p = 0.0006), supporting previous associations of IL12B with TB susceptibility. We also observed significant interaction between a single nucleotide polymorphism (SNP) in SLC11A1 and the L4-Ugandan lineage in both cohorts (rs17235409, meta p = 0.0002). Interestingly, the presence of the L4-Uganda lineage in the presence of the ancestral human allele associated with more severe disease. These findings demonstrate that IL12B is associated with severity of TB in addition to susceptibility, and that the association between TB severity and human genetics can be due to an interaction between genes in the two species, consistent with host-pathogen coevolution in TB.

## Author summary

Susceptibility to tuberculosis (TB) is affected by genetic variation in both the human host and the causative bacterium, *Mycobacterium tuberculosis*. However, prior studies of the genetics of each species have not explained a large part of TB risk. The possibility exists that risk can be better estimated from patterns of variation in the two species as a unit, such that some combinations provide increased risk, or in the presence of TB, increased disease severity. We hypothesized that alleles in the two species that have co-existed for long periods are more likely to reduce disease severity so as to promote prolonged co-occurrence. We tested this by studying TB severity in two patient cohorts from Uganda for which paired MTB-human DNA were available. We examined severity, as measured by the Bandim TBscore, and assessed whether there was an interaction between MTB lineage and SNPs in the host with this metric. Our results indicate that the most recent TB lineage (L4.6/Uganda) when found together with an ancestral allele in SLC11A1 resulted in more severe disease. This finding is consistent with the conclusion that MTB and human have coevolved to modulate TB severity.

## Introduction

Pulmonary tuberculosis (TB), a respiratory disease caused by *Mycobacterium tuberculosis* (MTB) infection, creates a significant public health burden worldwide, with 10 million incident cases and an estimated 1.64 million deaths in 2017 [1]. Susceptibility to pulmonary TB can be influenced by human genetic variation with both candidate gene and genome-wide studies having identified variants that affect risk of disease [2–8]. However, to our knowledge there has been only one study characterizing TB severity as a quantitative trait and examining genetic associations with this trait [9].

There is evidence that MTB genetic variation as delineated by phylogenetic lineage can independently affect TB sequelae and manifestations of disease severity [10–13]. There are many species within the *Mycobacterium* genus. The *Mycobacterium tuberculosis* complex, which causes most human disease, is classified into seven major lineages with different geographical boundaries and timelines of human exposure [14]. Some of these are ancient (L1, L5, L6, L7) while others are modern (L2, L3, and L4). Generally, ancient lineages of MTB are less virulent than the modern ones, lending some weight to the hypothesis that the emergence of newly evolved lineages leads to more virulent disease, a phenomenon referred to as disrupted coevolution [10, 15–19]. In the case of TB disease, virulence is highly correlated with the presence and severity of active TB symptoms as they are necessary for the Mycobacteria to spread [18]. Lineage 4 (L4), Lineage 2 (L2), and Lineage 3 (L3) are the most common, and L4 is the most widespread worldwide. L4 is thought to have originated in Europe prior to its global spread. L3 is mostly found in the Middle East, India, and East Africa, while L2 is found predominantly in East Asia. Recent work has shown that several sub-lineages of L4 are more recently evolved than the major L4 lineage, although dates for these events have been difficult to determine. Over time, at least 10 genetically distinct sub-lineages of L4 arose in highly restricted geographic ranges as compared to the seven major lineages. Important to our study, there is a sub-lineage found solely in Uganda and neighboring countries known as the L4.6/Uganda sub-lineage. L4.6 appears to be the most common lineage among active TB cases in our cohort [15–17, 20]. This sub-lineage has been shown to have highly conserved T-cell epitopes (i.e. a lower proportion of variable epitopes) and a much smaller geographic range than non-specialized lineages, indicating that it may be adapted to a specific host population(s) [14]. In most cases, the lineages we studied can be distinguished based on either single nucleotide polymorphisms (SNPs) or long sequence polymorphisms (LSPs) that have been identified as a “barcode” through whole genome sequencing [10]. The present study utilized both the LSP and SNP-based phylogeny from previously published and validated studies [10, 14–17, 20].

The theories of prudent exploitation and disrupted coevolution suggest that long-term coexistence between the human genome and an MTBC lineage may decrease the severity of disease and the presence of newly evolved or introduced strains may cause more severe disease [19, 21–23]. While this has not been shown on a population level, the possibility of coevolution between humans and MTBC has previously been suggested as an important area of research. Suspicion of coevolution is based on a 70,000 year co-existence with humans, the observation that modern lineages of MTB are more virulent than ancient ones, and that certain lineages and sub-lineages of MTBC appear to be adapted to specific human populations [18]. Consistent with the coevolution hypothesis, a study of TB transmission in San Francisco showed that TB transmission was most likely to occur among its sympatric host population despite mixed exposure [24].

Coevolution implies distinct historical and geographic variation in the prevalence of MTB lineages that would allow the host and pathogen to adapt to each other, thereby enabling coordinated evolution between host and MTB genotypes. Coevolution can be demonstrated when there are reciprocal effects of the two traits (both pathogen and host) on fitness. Operationally, this can be shown via population genetic analyses when the outcome (fitness) depends on the interaction of the two traits involved [25]. In this study, our outcome correlates with survival and therefore, reproductive fitness. If local adaptation exists, it should be possible to measure disease risk or severity in terms of human–MTB coevolution as a result of historical coexistence [25, 26]. This can be statistically assessed by testing for interaction between the genetics of the host and MTB lineage [19, 27]. If this is the case, it would provide evidence to support the theory of prudent exploitation/coevolution in which infection does not necessarily lead to active disease and may cause less virulent disease, when present [22, 28]. In fact, most people exposed to MTB do not progress to active disease [29]. Under the coevolution model, a newly divergent MTB lineage (one that has not historically co-existed with the population in question) is expected to cause more severe disease [23]. The potential for human-MTB coevolution has been explored in human and model systems, but studies have not yet identified an effect at the population level [3, 4, 10, 13, 30–32]. Although coevolution may affect the likelihood of developing active disease, to study coevolution in practice, it is necessary to study severity in cases only, as we cannot deduce the MTB lineage(s) to which unaffected individuals have been exposed with certainty. Even within a household, the strain to which an individual is predominantly exposed may not match that of the index case, since community exposure is thought to be the major source of exposure [33].

Prior evidence supports the role of host genotype and MTBC lineage in TB disease, but it is unclear whether there are interactions between the genomes of the two species. Studies examining the interaction between host genotype and MTB lineage are sparse, especially those examining severity as an outcome. Because uninfected or latent individuals cannot be examined for MTB lineage, all previous studies of host-MTB genome interaction have been case-only studies that examine association between the lineage present and the host genotype, but this does not truly show interactions between the lineage and host genotype [30, 31, 34, 35]. While this information is valuable, these studies demonstrate that some lineages are more common among active TB cases with certain genotypes, which is not true interaction. Host-pathogen coevolution has been demonstrated in other organisms by examining a multiplicative interaction term between host and pathogen genotypes [19, 27]. *Helicobacter pylori* is the most notable example of this framework being employed to detect coevolution. Simply, where we have information about host population genetics and the evolutionary history of the pathogen, we can use effect modification or interaction in a statistical model as evidence for coevolution between the two species. In TB, this has been problematic, as interactions are best detected using severity or virulence on a continuous scale. To study TB severity, we have employed the Bandim TBscore that examines clinical severity using symptoms and clinical examination [36]. The Bandim score is predictive of mortality among TB patients who are receiving treatment for their illness and associates with procalcitonin and C-reactive protein, biomarkers of TB severity [37–40]. In the present study, this measure was used to assess severity of TB and the potential role of coevolution between MTB and humans. A distinct advantage of the TBScore is that it is based on simple measures of clinical parameters that can be determined in resource-limited environments where TB is most prevalent.

We assessed the role of human genetic variation and MTB lineage in TB severity considered singly, and also whether the two interact. We hypothesized that there is coevolution between host genotype and pathogen lineage and that this can be shown through a significant statistical interaction between the host variants and MTB lineage. In particular, based on the theoretical argument for coevolution, we hypothesized that the more ancient lineage of MTB should have reduced severity in the presence of ancient host alleles, and that the derived lineage should result in more severe disease, especially in the presence of the ancient host alleles. We tested this model explicitly in our study. This approach will help us elucidate the degree to which the effects of lineage on severity are modified by and thus dependent on the genotype of the human host.

## Results

### Population characteristics

Of the 113 subjects in cohort 1, 51 (45.2%) were female, and 46 (40.7%) were HIV positive (Table 1). Subjects ranged from age 16 to 67 and the mean age was 29.74 with a standard deviation of 9.1 years. The mean TBscore was 5.86 with a standard deviation of 2.20. Of the 121 subjects in cohort 2, 51 (42.2%) were female, and 31 (25.6%) were HIV positive. Subjects ranged in age from 15 to 70 with a mean of 29.17 and a standard deviation of 8.9. The mean TBscore was 5.53 with standard deviation of 2.21. The two cohorts were similar in every aspect except for HIV positive status (Table 1). Thus, as stated in our statistical methods section, we controlled for HIV status in all of our regression models so that HIV status would not alter our conclusions.

**Table 1:** Cohort demographics and clinical measures.

Variable	Total	Cohort 1	Cohort 2	p*
Sample Size	234	113	121	-
Female	102 (43.6%)	51(45.2%)	51(42.2%)	0.65
HIV+	77(32.9%)	46 (40.7%)	31(25.6%)	0.014
Age, years	29.44 ± 8.9	29.74 ± 9.1	29.17 ± 8.9	0.62
Age range, years	15–70	16–67	15–70	-
TBscore	5.69 ± 2.21	5.86 ± 2.20	5.53 ± 2.21	0.25

Values are shown as N (%) or as mean ± SD.

*Comparisons between Cohort 1 and Cohort 2 were made using Pearson’s *χ*^2^ test and for continuous variables using Student’s t-test.

### Lineage and severity

**Fig 1:**
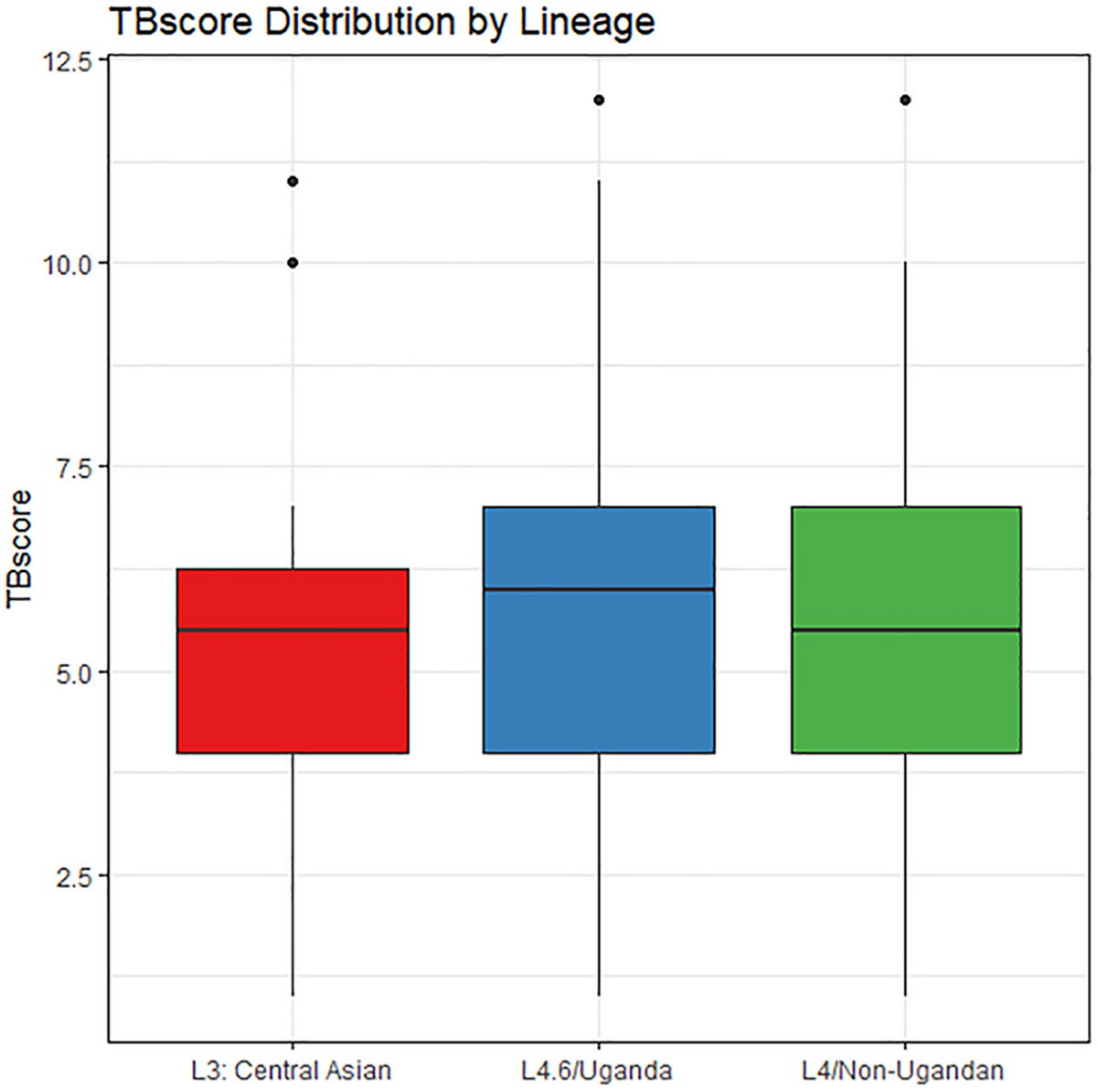
TBscore across lineages. Lines within each lineage show the median values. Mean TBscore (+/- S.D.) is 5.6 (+/- 2.2) for L4/Non-Ugandan; 5.4 (+/-2.4) for L3:Central Asia and 5.8 (+/-2.2) for L4.6/Uganda. These do not differ p = 0.71 (ANOVA).

There were 3 lineages detected in our study: L3: Central Asia, L4/Non-Ugandan, and L4.6/Uganda. L4.6/Uganda is the most prevalent lineage in our subjects (64.6%). The L4.6/Uganda sub-lineage is thought to have diverged from the L4 lineage and is only found in Uganda and neighboring countries. The L3 and L4 lineages have a longer history of human contact and are more geographically widespread [14–17, 20]. Severity of TB was not associated with lineage independently of human genotype (Fig 1, p = 0.71 by ANOVA), consistent with previous findings in a study of lineage and clinical characteristics of TB [20]. Comparisons of only the L4.6/Uganda to the other two lineages also indicated no differences in disease severity (p = 0.44). In addition, lineage distribution did not differ significantly between cohorts (p = 0.58; S1 Table). That lineage alone, without consideration of host genotype, does not predict severity is important to the interpretation of interaction models.

### Independent effects of MTB lineage and Human SNPs on TB score

Of the 403 SNPs tested, we observed significant association between multiple SNPs in *IL12B* and TBscore that was consistent across cohorts. Four SNPs were nominally significant (p ≤ 0.05) in Cohort 1 and all replicated (p < 0.05) in Cohort 2. A fifth SNP (rs3213094) was borderline significant in Cohort 1 (p = 0.06) but was significant in Cohort 2 and the combined data (p = 0.0009). The four SNPs that were significant in both sets showed perfect linkage disequilibrium (LD) (r^2^ ≥0.99 in all pairwise comparisons for the combined data (Table 2 and S1, S2 and S3 Figs); rs3213094 showed similarly high levels of LD with the other SNPs (S1 and S2 Figs). The SNP with the most significant combined p-value was rs3212227 (p = 0.0006). All of these SNPs had similar effect sizes (β values) in the linear regression model, ranging from 0.98 to 1.01 and four of the five passed a multiple testing threshold using FDR (q = 0.1, LD pruning r^2^ > 0.3). The presence of the homozygous ancestral genotype was associated with a one-point average increase in the TBscore, equivalent to one more clinically relevant symptom, meaning the derived allele is associated with lower severity. A score of 3 or greater is a predictor of 18 month mortality; therefore, even a one point increase is clinically meaningful in some contexts [36]. We also examined the relationship between rs3312227 and IFN-γ levels after stimulation with MTB culture filtrate in a subset of our samples (n = 73) [41, 42]. *IL12B* genotypes were significantly associated with IFN-γ levels post stimulation (p = 0.02; S2 Table). Notably, the genotype that associates with lower IFN-γ (GT or TT) associates with more severe disease as expected.

**Table 2:** Association between *IL12B* markers and TBscore, adjusted for HIV status.

			Cohort 1		Cohort 2		Combined		
Name	Alleles	Notation	β (95% CI)	p	β (95% CI)	P	β (95% CI)	p	FDR threshold ^1^
rs3212227	**T**/G	3' UTR	0.94 (0.08, 1.79)	0.03	1.05 (0.25, 1.85)	0.01	1.00 (0.44, 1.58)	0.0006	0.0005
rs3212219	**C**/A	Intron	0.89 (0.04, 1.74)	0.04	1.05 (0.25, 1.86)	0.01	0.98 (0.43, 1.56)	0.0007	0.0009
rs3212220	**C**/A	Intron	0.91 (0.01, 1.81)	0.05	1.05 (0.25, 1.87)	0.01	1.01 (0.40, 1.56)	0.0011	0.0023
rs3213094	**C**/T	Intron	0.84 (–0.02, 1.71)	0.06	1.05 (0.25, 1.85)	0.01	0.98 (0.41, 1.55)	0.0009	0.0019
rs6894567	**A**/G	Intron	1.00 (0.17–1.83)	0.02	0.90 (0.11–1.70)	0.03	0.97 (0.41–1.53)	0.0008	0.0014

All analyses coded using homozygous ancestral as 1 and one or more copies of the derived allele coded as 0.

The ancestral allele is bold in the table.

^1^ FDR corrected p value for q = 0.1 and LD corrected for r^2^ < 0.3 (213 SNPs)

While association with SNPs in other genes, *NOD1* and *STAT1*, was observed, strict replication was not obtained, as the same SNPs were not nominally significant (p < 0.05) across both samples (S3 Table). Different SNPs in these genes were significant in at least one of the cohorts.

### Examination of host-pathogen interaction

The interaction between SNP and lineage was assessed with a linear regression model that included a multiplicative term for SNP and lineage in addition to the first order effects of genotype and lineage (S4 Table). Interactions were considered significant only if p < 0.05 for the interaction term in both cohorts. Our analyses were focused on contrast between L4.6/Uganda and the other two lineages combined as it is the derived lineage that should associate with worse disease in the presence of ancestral human alleles, if coevolution is at play. A significant interaction between one SNP in *SLC11A1*, rs17235409, and L4.6/Uganda was observed in both cohorts. The effect for this interaction was in the same direction and of similar value in each cohort and showed a more significant association in the combined analysis (combined p = 0.000225) (Table 3), significant even after Bonferroni correction. We determined the Bonferroni correction using linkage disequilibrium structure to determine the number of independent SNPs; we performed 213 independent tests of statistical association. This yielded a threshold of 0.000235. Of note, rs17235409 is a non-synonymous exonic variant (D543N). Two other SNPs in *SLC11A1*(rs2279014 and rs13062) also show signs of interaction (S4 Table), although they do not pass multiple testing correction. These SNPs are not in strong LD with rs17235409 (S4, S5 and S6 Figs). According to ENSEMBL, this variant may either be a missense variant or lead to nonsense-mediated decay. Thus, the genetic variant in question is known to alter the amino acid sequence of the SLC11A1 protein or lead to changes in the amount of SLC11A1 protein being translated, showing that this variant is more likely to have a functional consequence. Furthermore, this SNP was not associated with TBscore when an interaction term was not included in the regression model (S3 Table). The interaction term associates with the biggest change in TBscore, and the interaction model explains more variance, which is almost ten times greater than that of the model that does not account for interaction, but includes the additive effects of both SNP and lineage (S5 Table). The effect size (β value) for the interaction in the combined analysis was 2.63. This means that the interaction between *SLC11A1* genotype and MTB lineage will lead to a 2.63 point increase on the severity scale, more than the effects of both genotype and lineage considered additively. The first order effects of both host genotype and MTB lineage were associated with lower severity while the interaction effect is associated with higher severity. While the overall effect of the complete model (SNP, lineage, and interaction term) does not appear to be great, the large effect size of the interaction indicates the extent to which human genotype modifies the effects of MTB lineage. In our data, individuals who eventually died during follow-up had on average a TBscore 2.5 greater than those who survived, a difference similar to the effect size of the interaction. There is a clear statistical interaction between genotype and lineage that is significant even after the most conservative approach to multiple testing corrections.

**Table 3:** Full regression model results for *SLC11A1* marker rs17235409.

	Cohort 1		Cohort 2		Combined	
	β (95% CI)	p	β (95% CI)	p	β (95% CI)	p
rs17235409 (β_3_)†	-2.53 (-4.29, -0.77)	0.0057	-1.85 (-3.29, -0.40)	0.014	-2.11 (-1.00, -3.23)	0.00026
L4.6/Ugandan(β_2_)	-1.43 (-3.36, 0.50)	0.15	-2.18 (-3.73, -0.63)	0.0067	-1.83 (-0.62, -3.03)	0.00331
HIV+ Status(β_1_)	0.30 (–0.51, 1.11)	0.47	-0.47 (-1.39, 0.44)	0.31	0.033 (-0.56, 0.63)	0.911
rs17235409*L4.6/Ugandan (combination of GG and L4.6/Ugandan) (β_4_)	2.36 (0.21, 4.50)	0.033	2.71(0.91, 4.51)	0.0039	2.63 (1.25, 4.00)	0.000225

rs17235409 is an exonic SNP at position 219259732 on chromosome 2. The ancestral allele is G and the derived is A.

Regression Model:

Y = β_0_ +β_1_X_1_ + β_2_X_2_ + β_3_X_3_ + β_4_ (X_2_X_3_) + ε

X_1_ = rs17235409, X_2_ = HIV+ Status, X_3_ = Ugandan Lineage.

†Model of inheritance (GG vs GA/AA as referent).

**Fig 2:**
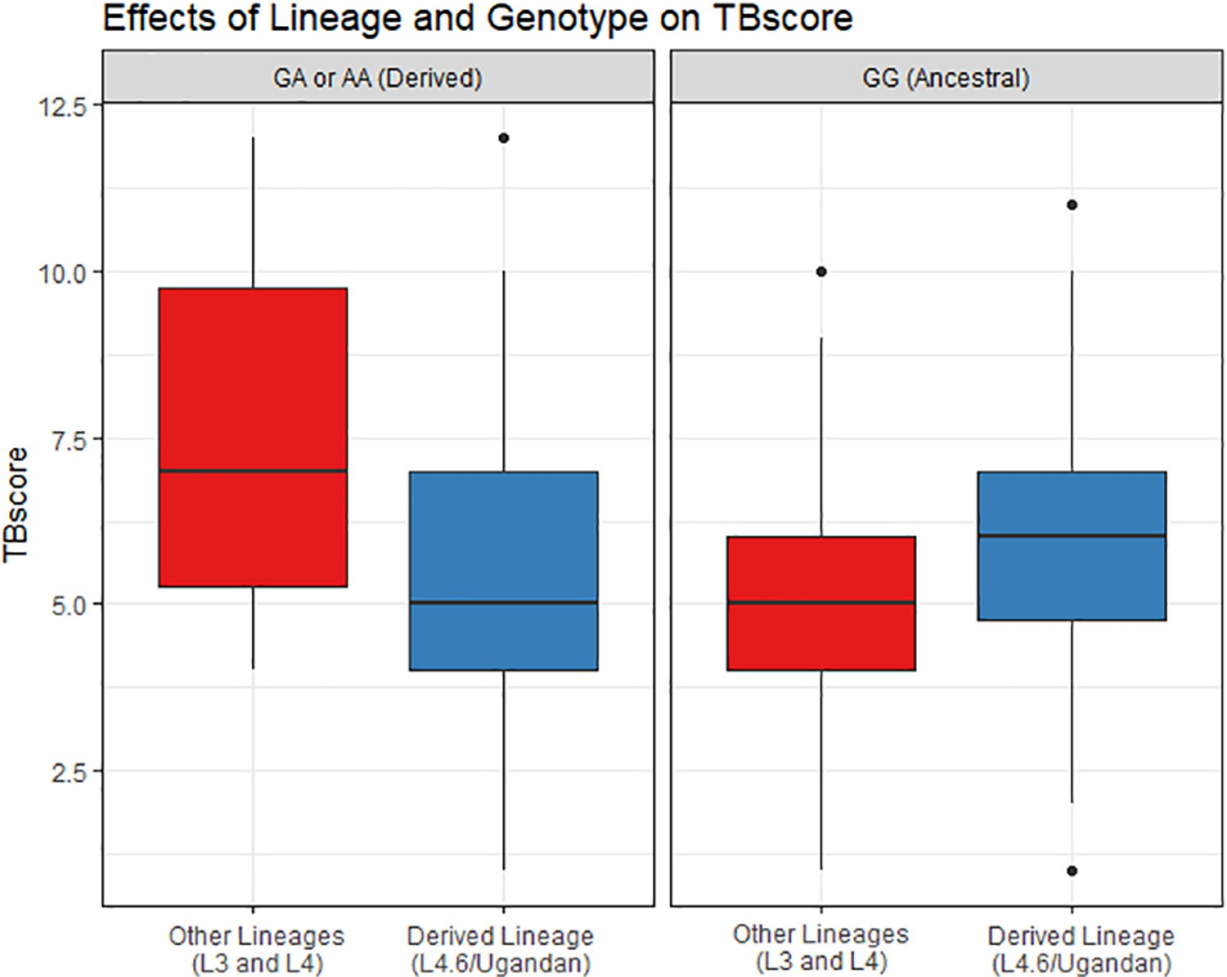
Effects of rs17235409 (SLC11A1) genotype and lineage on TBscore. The association of TB severity with lineage differs but only when stratified by genotype. TBscore is smaller in the Uganda lineage for individuals carrying a derived allele (GA or AA)(left panel) at *SLC11A1* but larger for the ancestral genotype (GG) carriers (right panel). These results show the underlying bases of the significant interaction between rs17235409 and MTB lineage (p = 0.00022) with genotype and lineage effect directions differing when examining stratified data.

To interpret our results in the context of coevolution, we must look at the direction of this interaction and understand the possible scenarios a subject could experience with respect to genotype and lineage in our model. Consider that there are four possible combinations of genotype and lineage in this analysis, holding HIV status constant. The combinations with the highest TBscore were: (1) the carriers of derived alleles (AA, GA) with the lineages that are more widespread and have had longer historical contact with humans (L3 and L4) and (2) the ancestral homozygous genotype (GG) with the more newly evolved Ugandan sub-lineage (L4.6). This is important as we observe the lowest average TBscore for the combination of ancestral allele and older lineages, i.e. the genotype and lineage that have historically co-existed. We observed the highest average TBscore for the combinations of genotype and lineage that have not historically co-existed. These findings support our model that coevolution between a lineage and genotype would associate with lower severity. For this SNP, the allele that associates with more severe disease in the interaction model is the ancestral human allele, G (Fig 2 and Table 3). In both cohorts, the simultaneous presence of both the ancestral human allele and the derived L4.6/Uganda sub-lineage was associated with increased TBscore, indicating that evolutionary histories of both species, taken together, affected disease severity. That the SNP associates only in the presence of an interaction term is indicative of coevolution between human variants and MTB lineage.

## Discussion

Our hypothesis is that coevolution between humans and *M*. *tuberculosis* in the same area may lead to less severe disease when host individuals possess the ancestral allele and are infected with a historically co-existing lineage of MTB [21]. Our observation of more severe TB when the human homozygous ancestral genotype (GG) was combined with the more recently divergent L4.6/Uganda MTB sub-lineage and less severe TB when this younger lineage is combined with a derived genotype is in line with the conclusion that disrupted coevolution increases disease severity. The L4.6/Uganda lineage, almost exclusively found in Uganda and neighboring countries, is the most recently diverged clade in our sample, and is therefore not the lineage Ugandans have historically been infected with. Therefore, we posit that individuals with ancestral Ugandan human genotypes may not be able to mount as effective an immune response as they have not historically been exposed to L4.6/Uganda, the derived lineage. Additionally, if an MTB lineage has evolved in the context of specific human standing variation, then we would hypothesize a derived lineage to associate with more severe disease, as we observed in Uganda, consistent with the hypothesis of prudent exploitation. That the lineages alone did not associate with severity is due to the fact that on average (across all human genotypes) the lineage does not affect TB, but it does trend in different directions in the two human genotype classes we analyzed.

In our two independent sets of subjects, we identified both marginal effects for TB severity in loci previously associated with TB disease (*IL12B*) and evidence for interactions between human SNPs in *SLC11A1* and MTB lineage. Of note, the *SLC11A1* SNP that interacted with MTB lineage did not associate with TBscore in the absence of interaction, indicating that the host genotype and the lineage of the pathogen are important only when considered together. These interactions in the absence of marginal effects have previously been considered a hallmark of coevolution [19, 27].

The genes we have analyzed in both the marginal analysis and interaction analysis were chosen because they are all biologically plausible as they produce end-products known to be important in the immune response to MTB and have been previously identified as modifiers of responses to MTB infection [3, 43]. *IL12B* is an important regulator of immune responses to MTB, including IFN-γ secretion. A previous epidemiologic study has suggested *IL12B* as a determinant of TB susceptibility in humans, and previous studies have shown associations with one of the same SNPs that we replicated in both sets (rs3212227) [8, 44]. Additionally, prior work has shown that IL12 pathway deficiency associates with mycobacterial diseases [45]. Lastly, murine models have shown that an IL12 gene knockout renders mice highly susceptible to MTB infection [46]. While *IL12B* has previously been shown to be important in TB, these studies did not examine severity. We believe that previous evidence in the context of susceptibility is consistent with our findings that *IL12B* is an important determinant of TB disease severity. However, it is important to consider the possibility that distinct biological processes are underlying each phenotype and thus this result is a novel finding that adds to previous literature about the putative role of *IL12B* in TB pathogenesis. Further, very few studies have examined clinical severity as a phenotype and it is important to expand this area of study.

*SLC11A1* (previously called *NRAMP1*), an important regulator of macrophage responses to MTB, has long been implicated in resistance to intracellular infections, has been shown to impact the bacillary load of BCG infected mice, and may have a role in mycobacterial susceptibility at a population level [5, 47, 48]. *SLC11A1* has been well characterized and is thought to code for a membrane-bound divalent cation transporter found exclusively in macrophages and polymorphonuclear cells that has pleiotropic effects on macrophage activation. It functions on the phagolyosomal surface in macrophages, regulating changes in iron transport and the transport of other cations in response to infection. Further, MTB expresses a membrane-bound cation transporter in the *SLC11* family that functions similarly to, and potentially in competition with, the homologous human transporter to modify the iron content of the phagosome environment [34, 47–49]. Iron is important for bacterial proliferation and competition for iron between host and pathogen has been well documented. Bacteria have previously been thought to evolve strategies for iron acquisition, further supporting the argument that *SLC11A1* could be a plausible candidate for coevolution between humans and MTB, although it has not been determined which MTB genes are involved [50–52]. *SLC11A1* polymorphisms have also been shown to associate with expression of IFN-γ, and the frequency of polymorphisms in *SLC11A1* are different among populations, including between Europeans and Africans [34, 47, 48]. Despite strong functional data indicating a biologically plausible role in the human response to TB, association studies of *SLC11A1* have shown inconsistent results with variants in this gene across global populations; analyses have shown that both the presence of statistical significance and the direction of effects vary between studies, both within and between populations [5, 53–65]. Specifically, an association between rs17235409, the specific SNP in the interaction term that we have replicated in both cohorts, and TB susceptibility has been previously published in five studies [55, 56, 61, 62, 64]. Of these five studies, two showed no association, one showed an association only among women below a certain age, and two showed a positive association with TB susceptibility. A recent study of susceptibility has also shown interaction between the Beijing strain of MTB and polymorphisms in *SLC11A1* [34]. That *SLC11A1* has a strong biological justification but variable results in prior human genetic analyses make it a strong candidate for a gene susceptible to coevolution in which human association is affected by MTB lineage.

Based on the current study, it is possible that the inability to find a consistent association between *SLC11A1* genotype and TB disease could possibly be affected by the unmeasured presence of different MTB lineages in prior studies that may have modified the association between *SLC11A1* and TB. While there may be other explanations for these findings, we think coevolution is the most likely reason for this interaction based on a well characterized function in the context of TB, the extent and variability of prior genetic epidemiological studies, and the fact that our statistical interaction results and predicted values are in line with previous literature studying coevolution. While these results do not definitively demonstrate coevolution, they do show clear effect modification and statistical interaction that is consistent with altered fitness, the hallmark of coevolution.

## Materials and methods

### Ethics statement

The study protocol was approved by the National HIV/AIDS Research Committee of Makerere University and the institutional review board at University Hospitals Cleveland Medical Center. Final clearance was given by the Uganda National Council for Science and Technology. All participants provided written informed consent.

### Study participants

TB cases, extensive clinical data, and MTB isolates were collected as part of the Kawempe Community Health Study in Kampala, Uganda [66] (S1 Text). We examined two cohorts (N = 113 and N = 121, respectively), independently collected in Kampala. All TB cases were culture-confirmed; additional details about ascertainment and clinical characterization are provided elsewhere [66]. The two cohorts differed in percentage of HIV positive individuals (Table 1); therefore, HIV status was used as a covariate in all of our regression models. Previous analyses of microsatellite data from these cohorts indicated no substantial population substructure [67]. Symptoms utilized in the TBscore were evaluated upon diagnosis.

### Bandim TBscore

The TBscore is based on five self-reported symptoms: cough, hemoptysis, dyspnea, chest pain, and night sweats, as well as six signs identified at examination: anemia, pulse > 90 beats/min, positive findings at lung auscultation, temperature > 37°C, body mass index (BMI) < 18 kg/m^2^, and mid upper arm circumference (MUAC) < 220 mm. Each of the 11 clinical variables contributes 1 point, while BMI and MUAC contribute an extra point if <16 kg/m 2 and < 200 mm, respectively; thus, the maximum TBscore is 13. In our study, we did not have data on MUAC, so we instead used lean and fat mass body composition data obtained using bioelectrical impedance analysis (BIA), as described previously [68]. Taken at the initiation of treatment, a TBscore of 8 or greater is associated with a 10% greater chance of eventual death within 8 months than a TBscore under 8 and a TBscore of 3 or higher is a significant predictor of mortality over 18 months of follow-up, even after adjusting for HIV status [36]. The TBscore also declines among patients who are successfully treated. Further, the TBscore associates with procalcitonin and C-reactive protein, which are biomarkers of granulomatous disease and inflammation, and with the Karnofsky Performance Status, which has been used extensively in multiple medical fields to quantify quality of life and ability to carry out activities of daily life [38, 39]. Thus, the Bandim TBscore is a valid clinical measure that provides meaningful value in its ability to assess patients’ severity as well as determining prognosis.

### Human genetic analysis

For our analysis of human host genetics, we examined 29 genes that affect innate and adaptive immune responses to TB chosen as part of a previous candidate gene study in Cohort 1. This candidate gene panel has been tested for association with TB but not with severity or TBscore [43]. Single nucleotide polymorphisms (SNPs) in these genes were genotyped on a custom Illumina GoldenGate microarray that only included these loci of interest. This analysis focused on genes in the Toll-like and Nod-like receptor families (*TLR1*, *TLR2*, *TLR4*, *TLR6*, *TLR9*, *TIRAP*, *TOLLIP*, *TICAM1/2*, *MyD88*, *NOD1*, *NOD2*), cytokines and their receptors expressed by macrophages *(TNF*, *TNFR1/2*, *IL1α/β*, *IL4*, *IL6*, *IL10*, *IL18*, *IL12A/B*, *IL12RB1/2*, *IFNG*, *IFNGR1/R2*), genes expressed by T-cells (*IFNG*, *IL4*, *IL12*, *STAT1*, *IL12RB1/2*, *IL10*) and key TB candidate genes (*SLC11A1*, *SLC6A3*). Haplotype tagging SNPs were selected to capture common genetic variation (minor allele frequency ≥ 5%) with strong coverage (linkage disequilibrium r^2^ ≥ 0.8) in any of the three African HapMap populations (YRI, LWK, MKK), based on previous analyses [69]. Many of these have previously been studied in animal, human, and macrophage models and are thought to be important in the human response to MTB infection [3–5, 70].

For Cohort 2, we used the Illumina HumanOmni5 microarray comprising 4,301,331 markers genome-wide, offering high genome wide coverage of common genetic variation even within African populations [71]. Genotype calling and quality control were performed as described in elsewhere [71]. Since the Cohort 2 data did not contain all the SNPs of interest from the Cohort 1 data, we used the Michigan Imputation server and protocols to impute SNPs [72, 73]. Low quality imputed SNPs (minimac r^2^ criterion < 0.5) were removed.

Only SNPs that had a call rate greater than 0.95 and MAF> 0.05 in both samples were used in the analysis. This resulted in a total of 403 eligible SNPs that were tested for association with TBscore and for interaction with MTB lineage (S3 Table). We examined all SNPs in Cohort 1, then attempted to replicate these in Cohort 2. As the two cohorts were similar to each other with respect to dependent and independent variables, we then combined the two cohorts into one and ran the analyses again on this combined cohort.

Because of the IRB restriction on the data from Uganda, individual level data are only available upon request from the Uganda Genetics of TB Data Access Committee by contacting Dr. Sudha Iyengar (ski@case.edu).

### MTB molecular analysis

MTB was isolated from sputum of each of these subjects and lineages were classified according to lineage-identifying SNPs using real-time PCR and validated with long sequence polymorphism (LSP) PCR [20]. Lineage was determined from three SNPs that accurately distinguish the L4.6 Uganda, L3, and L4 lineages, as previously described by Wampande and Gagneux [14–16]. The classifications delineated by these SNPs were then compared to previously established LSP based lineages to validate these distinctions. Lineage 4 (L4) is the most geographically widespread and has historically been present across most of the world [14, 17]. L4 has many derived sub-lineages that each exist only within specific regions and to which people in these regions have been exposed for a shorter time [14, 17]. In the context of this study setting, the relevant MTB lineages were Lineage 4 (referred to in this paper as L4/Non-Ugandan), Lineage 3 (L3 also known as Central Asian), and Lineage 4.6/Ugandan, which is a specialist sub-lineage of L4 that is only found in Uganda and the countries immediately surrounding it (S1 Table) [14–17, 20]. L4.6/Uganda is the most newly evolved of the three, a sub-lineage of the L4 generalist lineage, and is unique to this part of Africa [14, 17].

SNP and LSP-based phylogeny has been proven to be consistent in multiple studies of MTBC sub-lineages and the body of literature on MTBC lineages indicates that this is the ideal method for researching the L4 sub-lineages [15, 24, 74]. Low sequence variation and lack of horizontal transfer make SNPs and LSPs a method well-suited to distinguishing lineages and this approach has been previously validated and published [15, 20, 75].

### Statistical analysis

The analysis of the marginal (main) effects analysis was done by analyzing SNPs as the independent variable and the TBscore as the dependent variable in a separate linear regression equation for each SNP, adjusting for HIV status as a binary covariate. SNPs were analyzed such that 1 was equal to the homozygous ancestral genotype and 0 was equal to the presence of the derived allele, as determined by the ENSEMBL and RefSeq reference genomes. We also analyzed these SNPs using ordinal regression and Poisson regression equations to ensure our conclusions were robust to our decision to use linear regression. Analysis was performed and figures generated using R version 3.5.2.

For our interaction term between lineage and human genotype, we chose to operationalize lineage as a binary variable. Each subject is coded as 1 for the L4.6/Ugandan lineage or as 0, which encompasses the L4/Non-Ugandan and L3/Central Asian lineages together. As the L4/Non-Ugandan and L3/Central Asian lineages have a longer history of human contact and L4.6/Ugandan is a newer sub-lineage, this enables us to examine coevolution as we are contrasting a lineage which is more recently evolved, relative to the two older lineages (L3 and L4). As we would expect a longer historical co-existence to associate with lesser severity and the introduction of a newer sub-lineage to associate with greater severity, we have grouped the two older lineages together. This also affords us greater power to detect a difference than if we were to examine all 3 lineages independently.

We calculated a Bonferroni criterion for experiment-wide significance based on the number of SNPs analyzed that accounted for linkage disequilibrium. To calculate a Bonferroni correction for the significance threshold, we determined the linkage disequilibrium structure to determine the number of independent SNPs and therefore independent tests of statistical association to be 213 based on a linkage disequilibrium threshold of r^2^ = 0.3. This yielded a p-value threshold of 0.000235. (S1 Text).

## Supporting information

S1 Text Description of supplemental methods and supplemental citations. (DOCX)

S1 Table Distribution of MTB lineages in cohort 1, cohort 2, and combined cohorts. (DOCX)

S2 Table Association between IFN- γ levels and rs3212227 genotype using available data combined cohorts. (DOCX)

S3 Table Regression results for association between SNPs and TBscore, adjusted for HIV status. (XLSX)

S4 Table Regression results for association between SNP by lineage interactions and TBscore, adjusted for HIV status. (XLSX)

S5 Table Regression results for association between rs17235409 and TBscore, adjusted for HIV status and lineage. (DOCX)

S1 Fig Haploview LD Plot for IL12B in cohort 1. (TIFF)

S2 Fig Haploview LD Plot for IL12B in cohort 2. (TIF)

S3 Fig Haploview LD Plot for IL12B in combined dataset. (TIFF)

S4 Fig Haploview LD plot for SLC11A1 in cohort 1. (TIF)

S5 Fig Haploview LD plot for SLC11A1 in cohort 2. (TIFF)

S6 Fig Haploview LD plot for SLC11A1 in Combined Data. (TIFF)
