# Supplementary material for: Interaction between host genes and Mycobacterium tuberculosis lineage can affect tuberculosis severity: Evidence for coevolution?
Source: PLoS Genet. 2020 Apr 30;16(4):e1008728. doi: 10.1371/journal.pgen.1008728 (PMC7217476; doi:10.1371/journal.pgen.1008728)
Supplement: S1 Text — (DOCX) [file pgen.1008728.s001.docx]

**Study Population**

The study population includes participants from two independently recruited cohorts in Ugandan households. The first cohort of subjects, which will be referred to as Cohort 1, were enrolled in a household contact study that enrolled patients from 1995 to 1999 and from 2002 to 2012[1, 2]. Cohort 2, which is an independent cohort of subjects, were enrolled between 2002-2012. The original study protocol was reviewed and approved by the National HIV/AIDS Research Committee, The Uganda National Council of Science and Technology and the institutional review board at the University Hospitals Case Medical Center, Cleveland, OH, USA. Patients who presented to the study clinic with active pulmonary TB were enrolled as index cases. Diagnosis of pulmonary TB for the present study was confirmed based on isolation of MTB from clinical gastric or sputum samples. A total of 141 index cases from Cohort 1 and 135 from Cohort 2 met these criteria. We then excluded 19 cases from Cohort 1 and 9 from Cohort 2 because these participants were under 15 years old and our outcome measure was not valid to assess TB severity in patients this young. We further excluded 7 cases from the Cohort 1 and 3 from Cohort 2 who had missing information for the MTBlineage. After these exclusions there were 115 cases fromCohort 1 and 123 cases from Cohort 2 but two people were in both cohorts and were removed from both leaving 113 and 121 for analyses.

**Clinical Phenotyping**

The BandimTBscore is based on five self-reported symptoms: cough, hemoptysis, dyspnea, chest pain, and night sweats, as well as six signs identified at examination: anemia, pulse > 90 beats/min, positive findings at lung auscultation, temperature > 37 ° C, body mass index (BMI) < 18 kg/m^2^ , and mid upper arm circumference (MUAC) < 220 mm. Each of the 11 clinical variables contributes 1 point, while BMI and MUAC contribute an extra point if <16 kg/m 2 and < 200 mm, respectively; thus, the maximum score is 13[3-5]. In our study, we did not have data on MUAC, so we instead used lean and fat mass body composition data obtained using bioelectrical impedance analysis (BIA), as described elsewhere[6-8].

**Genotyping**

For Cohort 1, a custom Illumina GoldenGate 10k microarray was designed for a previous analysis of TB candidate genes[9]. This analysis focused on genes in the Toll-like and Nod-like receptor families (*TLR1, TLR2, TLR4, TLR6, TLR9, TIRAP, TOLLIP, TICAM1/2, MyD88, NOD1, NOD2*), cytokines and their receptors expressed by macrophages *(TNF, TNFR1/2, IL1α/β, IL4, IL6, IL10, IL18, IL12A/B, IL12RB1/2, IFNG, IFNGR1/R2*), genes expressed by T-cells (*IFNG, IL4, IL12, STAT1, IL12RB1/2, IL10*) and key TB candidate genes (*SLC11A1, SLC6A3*). Many of these have previously been studied in animal, human, and macrophage models and are thought to be important in the human response to MTB infection[10-13].

Haplotype tagging SNPs were selected to capture common genetic variation (minor allele frequency ≥ 5%) with strong coverage (linkage disequilibrium r^2^ ≥ 0.8) in any of the three African HapMap populations (YRI, LWK, MKK), based on previous analyses[14]. Tag SNPs were identified using Genome Variation Server (GVS) (http://gvs.gs.washington.edu/GVS137/index.jsp). Genotyping was conducted using the Illumina iSelect platform. Once SNPs were selected using GVS, their availability on the iSelect platform was verified; if a specific SNP was not available on iSelect, a nearby SNP was selected to replace it. Genotype calling and quality control was performed using Genome Studio, filtering the SNPs by call frequency, replicate errors, and clustering quality. Family relationships were corrected and resolved where needed, including defining subfamilies of first-degree relatives within households.

For Cohort 2, we used the Illumina HumanOmni5 microarray comprising 4,301,331 markers genome wide,offering high genome wide coverage of common genetic variation even within African populations[15]. Genotype calling and quality control were performed as described in a previous publication[15].

**Imputation**

Since theCohort 2 data cohort did not contain all the SNPs of interest from the Cohort 1 data, we used the Michigan Imputation server and protocols to impute SNPs[16, 17]. Low quality imputed SNPs (minimac r^2^ criterion < 0.5) were removed. Only SNPs that had a call rate greater than 0.95 in both samples were used in the analysis. This resulted in a total of 403 eligible SNPs. For the marginal analyses, 3 of the 403 SNPs returned an error message. For the interaction analyses in Cohort 1, 3 of 403 SNPs returned an error. In Cohort 2,8 SNPs returned an error. 393 SNPs have results for the interaction analyses in both data cohorts (did not return any error messages). These errors results from a MAF<0.05.

**Lineages**

Mycobacterium tuberculosis (MTB) has distinct genetic lineages historically associated with different regions of the world. MTB isolates were obtained from study participants and real time PCR was performed to genotype three SNPs used to classify MTB into lineages, as previously described by Gagneux et al. [18, 19]. This sample of Ugandan subjects comprised three lineages: L4.6/Uganda, L4/Non-Ugandan, and L3-Central Asian, as described in the main text.

**Statistical Analysis**

All analysis was performed using R version 3.5.2. The association between SNPs and the Bandim TBscore and the interaction between SNPs and MTB lineage was assessed using linear regression models. We assessed the relationship between SNPs and TB severity on the Bandim TB scale. Each SNP was the independent variable in a separate regression equation and were coded using a dominant model. The analysis included HIV status as a covariate. Nominal significance was defined as p<0.05, and replication was based on the same SNP having p<0.05 with effect in the same direction in both cohorts.

We analyzed the interaction between SNP and Ugandan lineage by creating multiplicative interaction terms for SNP and Ugandan lineage in a linear regression model. All analysis was done in R. This analysis also included HIV status as a covariate. Because of small sample sizes we did not perform similar analyses for the L4/Non-Ugandan or L3:Central Asian lineages. All SNPs from Cohort 1 were analyzed in Cohort 2. P-values below 0.05 for the interaction term in both datasets with coefficients in the same direction in both cohorts (both positive or both negative) were considered to be significant results. We then ran a combined analysis to generate meta P-values and the threshold for significance was corrected using a Bonferroni correction that is adjusted for linkage disequilibrium (LD) within the dataset by finding the equivalent number of independent tests of association. This adjustment is necessary for SNPs that are imputed using LD structure as many of the tests being run are correlated[20]. The Bonferroni correction yielded a threshold of p=0.00023 for our 403 SNPs, equivalent to 371 independent tests. For our predictions of the count of symptoms, we created a Poisson model of the interaction between rs17235409 and lineage that adjusted for HIV status as a covariate. We then used the predict function in R and rounded the predictions to generate counts of symptoms for each of the four combinations of lineage and rs17235409 genotype (Supplemental Table 4).

1. Guwatudde D, Nakakeeto M, Jones-Lopez EC, Maganda A, Chiunda A, Mugerwa RD, et al. Tuberculosis in household contacts of infectious cases in Kampala, Uganda. American journal of epidemiology. 2003;158(9):887-98. Epub 2003/10/31. PubMed PMID: 14585767; PubMed Central PMCID: PMCPMC2869090.

2. Stein CM, Zalwango S, Malone LL, Thiel B, Mupere E, Nsereko M, et al. Resistance and Susceptibility to Mycobacterium tuberculosis Infection and Disease in Tuberculosis Households in Kampala, Uganda. American journal of epidemiology. 2018;187(7):1477-89. Epub 2018/01/06. doi: 10.1093/aje/kwx380. PubMed PMID: 29304247; PubMed Central PMCID: PMCPMC6031055.

3. Rudolf F. The Bandim TBscore--reliability, further development, and evaluation of potential uses. Global health action. 2014;7:24303. Epub 2014/05/27. doi: 10.3402/gha.v7.24303. PubMed PMID: 24857613; PubMed Central PMCID: PMCPMC4032506.

4. Rudolf F, Joaquim LC, Vieira C, Bjerregaard-Andersen M, Andersen A, Erlandsen M, et al. The Bandim tuberculosis score: reliability and comparison with the Karnofsky performance score. Scandinavian journal of infectious diseases. 2013;45(4):256-64. Epub 2012/11/02. doi: 10.3109/00365548.2012.731077. PubMed PMID: 23113626.

5. Wejse C, Gustafson P, Nielsen J, Gomes VF, Aaby P, Andersen PL, et al. TBscore: Signs and symptoms from tuberculosis patients in a low-resource setting have predictive value and may be used to assess clinical course. Scandinavian journal of infectious diseases. 2008;40(2):111-20. Epub 2007/09/14. doi: 10.1080/00365540701558698. PubMed PMID: 17852907.

6. Fluegge K, Malone LL, Nsereko M, Okware B, Wejse C, Kisingo H, et al. Impact of geographic distance on appraisal delay for active TB treatment seeking in Uganda: a network analysis of the Kawempe Community Health Cohort Study. BMC public health. 2018;18(1):798. Epub 2018/06/27. doi: 10.1186/s12889-018-5648-6. PubMed PMID: 29940918; PubMed Central PMCID: PMCPMC6019214.

7. Mupere E, Malone L, Zalwango S, Chiunda A, Okwera A, Parraga I, et al. Lean tissue mass wasting is associated with increased risk of mortality among women with pulmonary tuberculosis in urban Uganda. Annals of epidemiology. 2012;22(7):466-73. Epub 2012/05/12. doi: 10.1016/j.annepidem.2012.04.007. PubMed PMID: 22575813; PubMed Central PMCID: PMCPMC3377556.

8. Mupere E, Malone L, Zalwango S, Okwera A, Nsereko M, Tisch DJ, et al. Wasting among Uganda men with pulmonary tuberculosis is associated with linear regain in lean tissue mass during and after treatment in contrast to women with wasting who regain fat tissue mass: prospective cohort study. BMC infectious diseases. 2014;14:24. Epub 2014/01/15. doi: 10.1186/1471-2334-14-24. PubMed PMID: 24410970; PubMed Central PMCID: PMCPMC3922730.

9. Hall NB, Igo RP, Jr., Malone LL, Truitt B, Schnell A, Tao L, et al. Polymorphisms in TICAM2 and IL1B are associated with TB. Genes and immunity. 2015;16(2):127-33. Epub 2014/12/19. doi: 10.1038/gene.2014.77. PubMed PMID: 25521228; PubMed Central PMCID: PMCPMC4352113.

10. Hoal EG, Dippenaar A, Kinnear C, van Helden PD, Moller M. The arms race between man and Mycobacterium tuberculosis: Time to regroup. Infection, genetics and evolution : journal of molecular epidemiology and evolutionary genetics in infectious diseases. 2017. Epub 2017/08/28. doi: 10.1016/j.meegid.2017.08.021. PubMed PMID: 28843547.

11. Orlova M, Schurr E. Human Genomics of Mycobacterium tuberculosis Infection and Disease. Current genetic medicine reports. 2017;5(3):125-31. Epub 2017/07/25. doi: 10.1007/s40142-017-0124-7. PubMed PMID: 29201558.

12. Stein CM, Sausville L, Wejse C, Sobota RS, Zetola NM, Hill PC, et al. Genomics of human pulmonary tuberculosis: from genes to pathways. Current genetic medicine reports. 2017;5(4):149-66. Epub 2017/10/12. doi: 10.1007/s40142-017-0130-9. PubMed PMID: 29805915.

13. Wiens KE, Ernst JD. The Mechanism for Type I Interferon Induction by Mycobacterium tuberculosis is Bacterial Strain-Dependent. PLoS pathogens. 2016;12(8):e1005809. Epub 2016/08/09. doi: 10.1371/journal.ppat.1005809. PubMed PMID: 27500737; PubMed Central PMCID: PMCPMC4976988.

14. Baker AR, Qiu F, Randhawa AK, Horne DJ, Adams MD, Shey M, et al. Genetic variation in TLR genes in Ugandan and South African populations and comparison with HapMap data. PLoS One. 2012;7(10):e47597. Epub 2012/11/01. doi: 10.1371/journal.pone.0047597. PubMed PMID: 23112821; PubMed Central PMCID: PMCPMC3480404.

15. Igo RP, Jr., Hall NB, Malone LL, Hall JB, Truitt B, Qiu F, et al. Fine-mapping analysis of a chromosome 2 region linked to resistance to Mycobacterium tuberculosis infection in Uganda reveals potential regulatory variants. Genes and immunity. 2018. Epub 2018/08/14. doi: 10.1038/s41435-018-0040-1. PubMed PMID: 30100616.

16. Das S, Forer L, Schonherr S, Sidore C, Locke AE, Kwong A, et al. Next-generation genotype imputation service and methods. Nature genetics. 2016;48(10):1284-7. Epub 2016/08/30. doi: 10.1038/ng.3656. PubMed PMID: 27571263; PubMed Central PMCID: PMCPMC5157836.

17. McCarthy S, Das S, Kretzschmar W, Delaneau O, Wood AR, Teumer A, et al. A reference panel of 64,976 haplotypes for genotype imputation. Nature genetics. 2016;48(10):1279-83. Epub 2016/08/23. doi: 10.1038/ng.3643. PubMed PMID: 27548312; PubMed Central PMCID: PMCPMC5388176.

18. Stucki D, Brites D, Jeljeli L, Coscolla M, Liu Q, Trauner A, et al. Mycobacterium tuberculosis lineage 4 comprises globally distributed and geographically restricted sublineages. Nature genetics. 2016;48(12):1535-43. Epub 2016/11/01. doi: 10.1038/ng.3704. PubMed PMID: 27798628; PubMed Central PMCID: PMCPMC5238942.

19. Wampande EM, Mupere E, Debanne SM, Asiimwe BB, Nsereko M, Mayanja H, et al. Long-term dominance of Mycobacterium tuberculosis Uganda family in peri-urban Kampala-Uganda is not associated with cavitary disease. BMC infectious diseases. 2013;13:484. Epub 2013/10/19. doi: 10.1186/1471-2334-13-484. PubMed PMID: 24134504; PubMed Central PMCID: PMCPMC3853102.

20. Gao X. Multiple testing corrections for imputed SNPs. Genetic epidemiology. 2011;35(3):154-8. Epub 2011/01/19. doi: 10.1002/gepi.20563. PubMed PMID: 21254223.
