# Supplementary material for: Interaction between host genes and Mycobacterium tuberculosis lineage can affect tuberculosis severity: Evidence for coevolution?
Source: PLoS Genet. 2020 Apr 30;16(4):e1008728. doi: 10.1371/journal.pgen.1008728 (PMC7217476; doi:10.1371/journal.pgen.1008728)
Supplement: S1 Table — (DOCX) [file pgen.1008728.s002.docx]

**S1 Table. Distribution of Lineages in each cohort**

|  | Cohort 1 | Cohort 2 | Total | p |
| --- | --- | --- | --- | --- |
| L4-Ugandan | 73 (64.6) | 73 (60.3) | 146 (62.4) |  |
| L4-Non-Ugandan | 29 (25.7) | 31 (25.6) | 60 (25.6) |  |
| L3-Central Asian | 11 (9.7) | 17 (14.0) | 28 (12.0) |  |
| Total | 113 | 121 | 234 | 0.58 |

Figures are shown as N (%). P-value is from a χ^2^ test of independence
