# Supplementary material for: Interaction between host genes and Mycobacterium tuberculosis lineage can affect tuberculosis severity: Evidence for coevolution?
Source: PLoS Genet. 2020 Apr 30;16(4):e1008728. doi: 10.1371/journal.pgen.1008728 (PMC7217476; doi:10.1371/journal.pgen.1008728)
Supplement: S2 Table — (DOCX) [file pgen.1008728.s003.docx]

**S2 Table. IFN-γ analysis**

|  | **regression of rs3212227 on IFNγ levels after stimulation with 10 micrograms culture filtrate** |
| --- | --- |
| **p-value** | 0.02 |
| **β** | -1.45 |
| **Δ(mean) IFNg levels between rs3212227 genotypes (GT/TT has higher severity and lower IFNg levels. GG is reference)** | -147.4 IU/ml |

Using log transformed linear regression, we found that rs3212227, an IL12B SNP, is associated with IFN- γ levels in the 73 patients for which this data were available. The genotype that associates with lower IFN-γ (GT or TT) associates with more severe disease (higher TBscore).
