# Supplementary material for: Interaction between host genes and Mycobacterium tuberculosis lineage can affect tuberculosis severity: Evidence for coevolution?
Source: PLoS Genet. 2020 Apr 30;16(4):e1008728. doi: 10.1371/journal.pgen.1008728 (PMC7217476; doi:10.1371/journal.pgen.1008728)
Supplement: S5 Table — (DOCX) [file pgen.1008728.s006.docx]

**S5 Table. Regression model containing only additive effects of lineage and rs17235409 genotype**

|  | **Combined** | |
| --- | --- | --- |
|  | **β (95% CI)** | **p** |
| rs17235409 | -0.37 (-1.04, 0.30) | 0.273 |
| L4.6/Ugandan | 0.20 (-0.39, 0.80) | 0.506 |
| HIV+ Status | -0.054 (-0.67, 0.56) | 0.863 |

*rs17235409 coded as 1 for GG (ancestral) and 0 for GA/AA (derived). R^2^ = 0.008 for this model, compared to R^2^ = 0.07 for the model including the interaction term.
