# Supplementary figures and images for: Interaction between host genes and Mycobacterium tuberculosis lineage can affect tuberculosis severity: Evidence for coevolution?
Source: PLoS Genet. 2020 Apr 30;16(4):e1008728. doi: 10.1371/journal.pgen.1008728 (PMC7217476; doi:10.1371/journal.pgen.1008728)

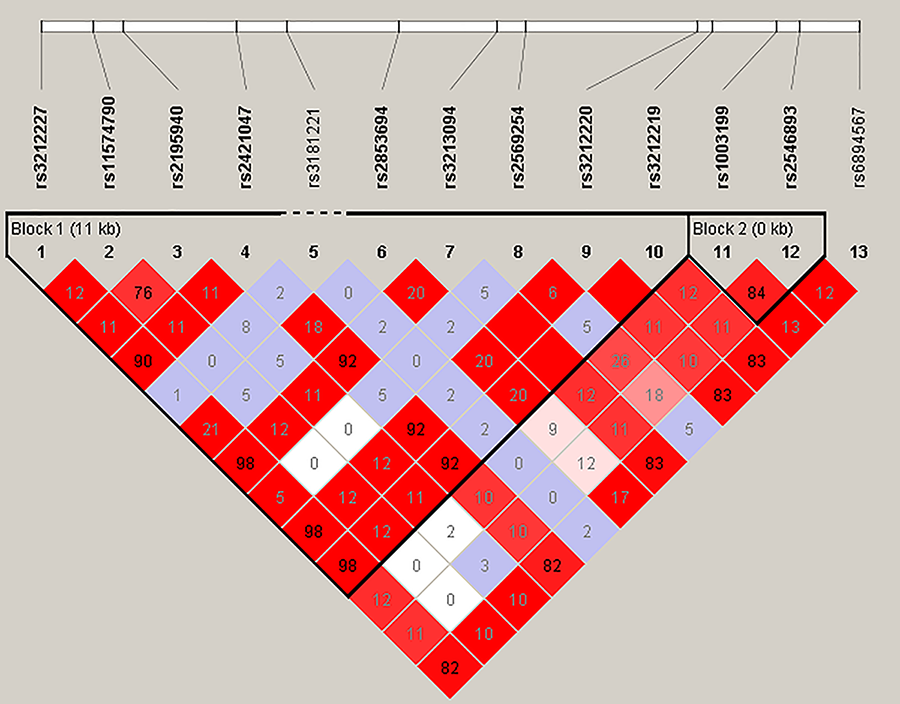

Supplement: S1 Fig — (TIFF) [file pgen.1008728.s007.tiff]

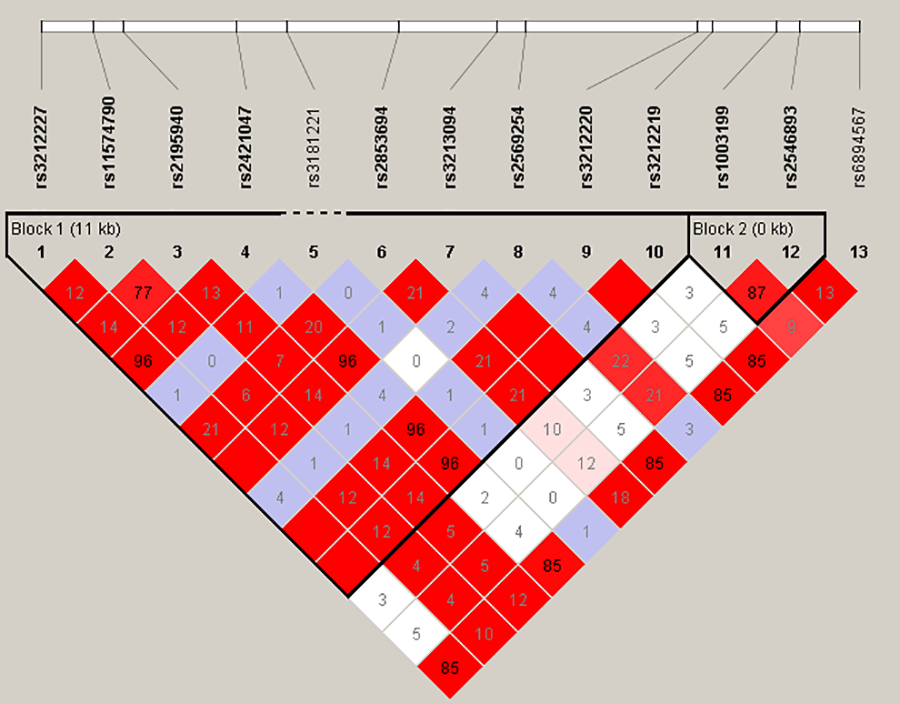

Supplement: S2 Fig — (TIF) [file pgen.1008728.s008.tif]

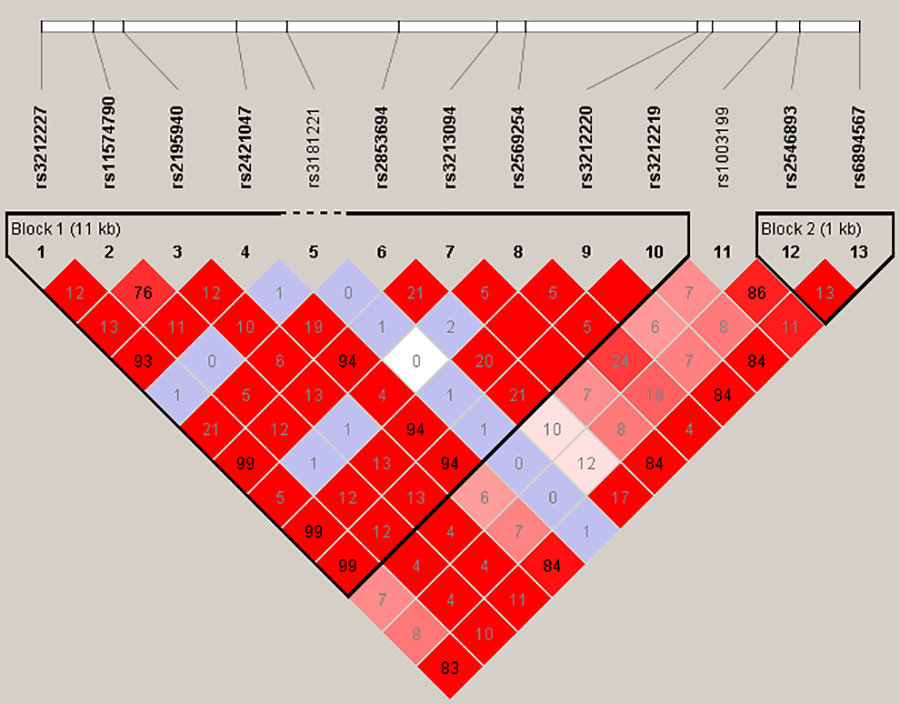

Supplement: S3 Fig — (TIFF) [file pgen.1008728.s009.tiff]

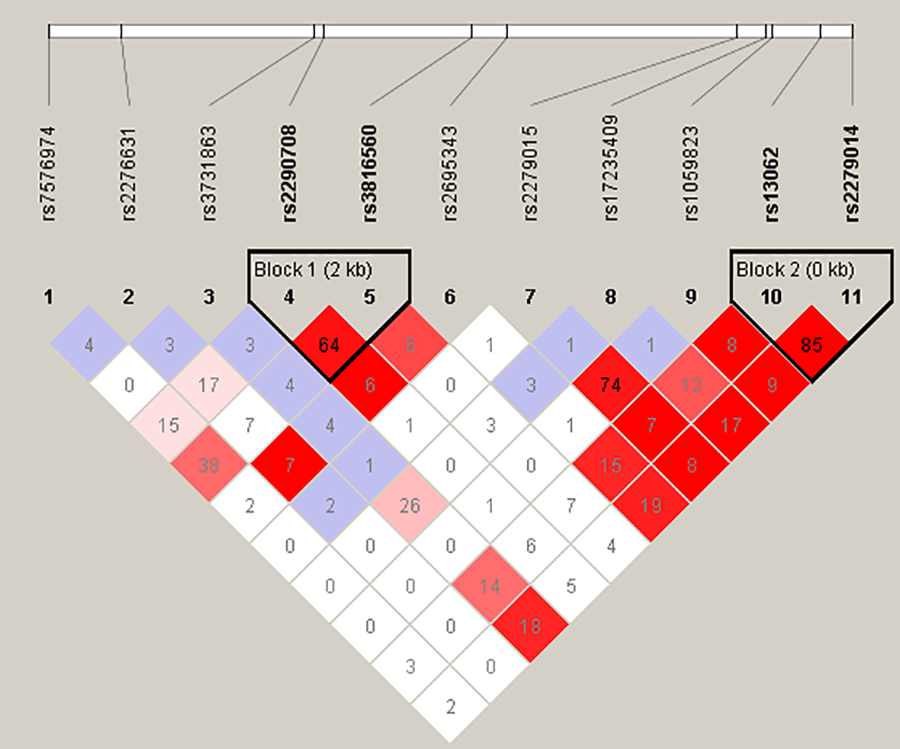

Supplement: S4 Fig — (TIF) [file pgen.1008728.s010.tif]

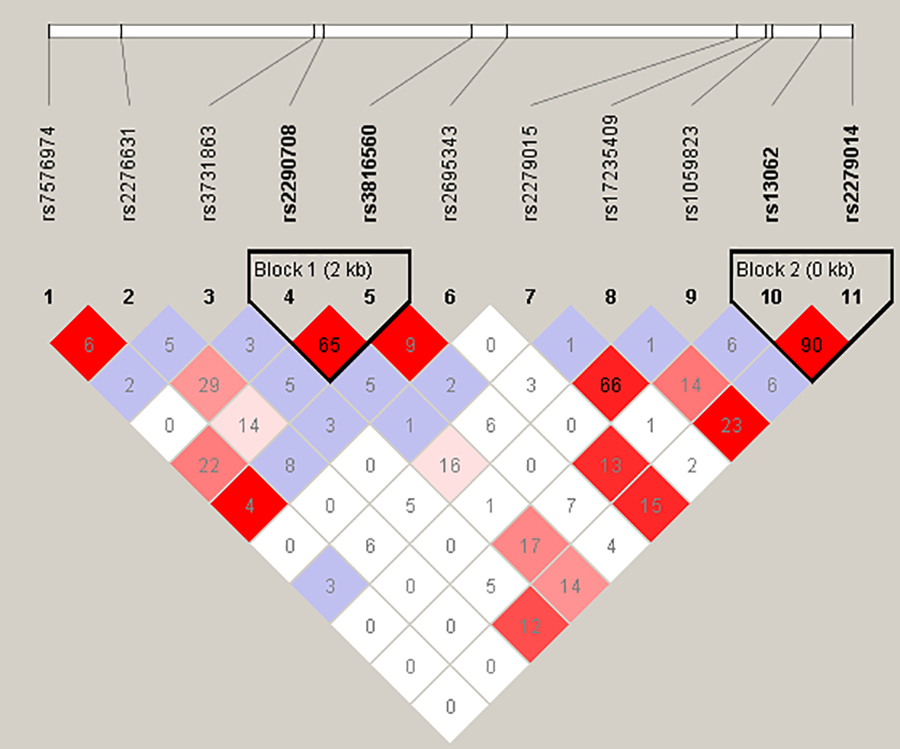

Supplement: S5 Fig — (TIFF) [file pgen.1008728.s011.tiff]

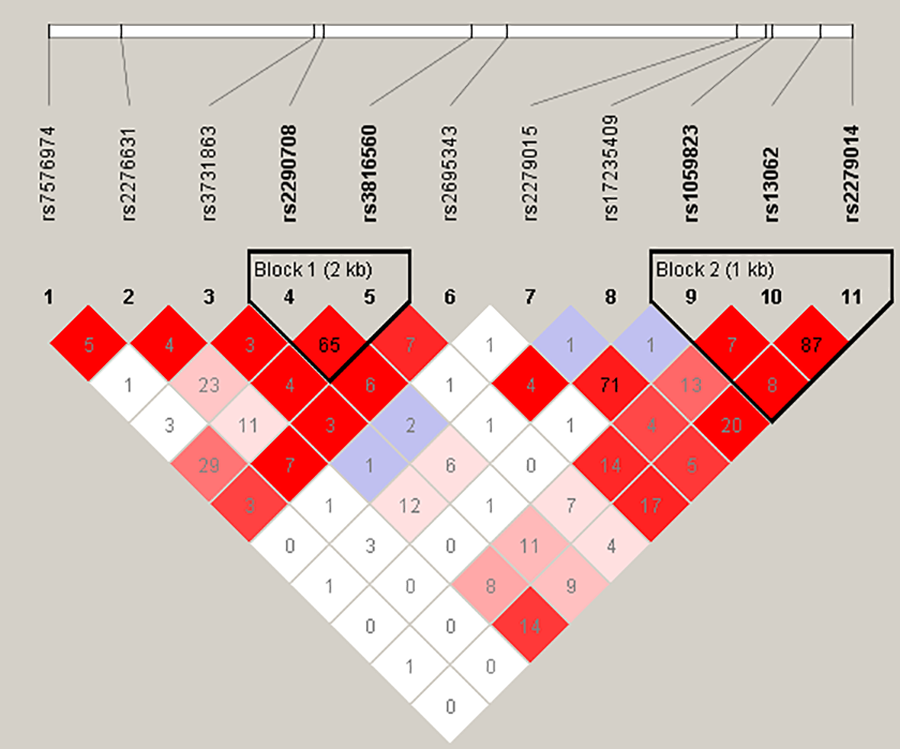

Supplement: S6 Fig — (TIFF) [file pgen.1008728.s012.tiff]
